# Outcomes of Air Versus Saline-filled Breast Expanders: A Systematic Review and Meta-analysis

**DOI:** 10.1007/s00266-025-04918-5

**Published:** 2025-05-23

**Authors:** Mario Alessandri Bonetti, Eleonora Bulgarelli, Elisa Dolfato, Gaia Ghiringhelli, Simone Catapano, Riccardo Carbonaro, Francesco Borelli, Andrea Lisa, Francesca De Lorenzi, Luca Vaienti

**Affiliations:** 1 Department of Plastic Surgery, University of Milan, Via Festa del Perdono 7, 20122 Milan, Italy; 2 Department of Plastic Surgery, IRCCS European Institute of Oncology, Via Ripamonti 435, 20141 Milan, Italy; 3 Department of Biomedical Sciences, Humanitas University, Humanitas University, Via Rita Levi Montalcini 4, Pieve Emanuele, 20090 Milan, Italy; 4 PhD Program in Applied Medical-Surgical Sciences - Department of Surgical Sciences, University of Rome “Tor Vergata”, Viale Oxford 81, 00133 Rome, Italy

**Keywords:** Breast, Implant, Expander, Cancer, Reconstruction

## Abstract

**Background:**

Among the possible implant-based reconstructive strategies, the two-stage tissue expander-to-implant procedure is one of the most common options in patients not ideal candidate to direct-to-implant reconstruction. Recently, other filling options such as air or carbon dioxide (CO2) have been reported as alternative fill media than saline for tissue expansion. The aim of this systematic review was to qualitatively and quantitatively synthetize the available evidence on the topic.

**Methods:**

A systematic review and meta-analysis were conducted, and they were reported according to PRISMA guidelines. PubMed, Embase, and Cochrane Library databases were accessed. Only studies with a control group were included. Risk ratios for complications were assessed between breast tissue expanders filled with saline versus air. MINORS criteria were used for bias assessment.

**Results:**

Nine studies met inclusion and exclusion criteria and were included. They encompassed a total of 1954 patients and 3243 breasts. Pooled risk ratios in air-filled compared to saline-filled breast expanders were calculated: total complications 0.92 [95% CI: 0.67; 1.27, *p*=0.53], mastectomy flap necrosis 0.86 [95% CI: 0.65; 1.12, *p*=0.26], hematoma 1.07 [95% CI: 0.63; 1.84, *p*=0.80], seroma 1.26 [95% CI: 0.91; 1.76, *p*=0.16], infection 0.80 [95% CI: 0.61; 1.04, *p*=0.09], extrusion 1.38 [95% CI: 0.82; 2.32, *p*=0.23], readmission 0.96 [95% CI: 0.58; 1.60, *p*=0.88]. The mean difference in days needed to achieve final expansion between air-filled and saline-filled breast expanders was -27.59 [95% CI: -46.42; -8.78, *p*=0.004].

**Conclusion:**

Air-filled expanders represent an alternative reconstructive option in the field of two-stage breast reconstruction. Despite limited by the only initial available evidence, they appeared to be safe and associated with a similar risk of complications compared to saline-filled expanders. However, they may enable faster postoperative expansion and fewer outpatient expansion visits compared to saline-filled expanders.

**Level of Evidence III:**

This journal requires that authors assign a level of evidence to each article. For a full description of these Evidence-Based Medicine ratings, please refer to the Table of Contents or the online Instructions to Authors  www.springer.com/00266.

**Supplementary Information:**

The online version contains supplementary material available at 10.1007/s00266-025-04918-5.

## Introduction

Breast cancer is the most common cancer in women [1, 2]. In the USA in 2023, the North American Cancer Society has estimated 297.790 new invasive breast cancer cases [1]. With increasing numbers of women diagnosed with and surviving breast cancer, the number of post-mastectomy breast reconstruction has continued to rise with 50–60% of patients electing to undergo reconstructive surgery [3]. Among the possible implant-based reconstructive strategies, the two-stage tissue expander-to-implant procedure is one of the most common options in patients not ideal candidate to direct-to-implant reconstruction [2, 4–6]. During the initial stage of the procedure, a saline-filled breast tissue expander is positioned either in the prepectoral or subpectoral plane. Subsequently, saline is gradually injected into the expander in an outpatient setting to promote skin expansion until achievement of the desired breast volume. During the second stage, the breast tissue expander is removed and the implant is then inserted. Breast tissue expanders have evolved in design and material since its introduction in the 1980 s by C. Radovan [6, 7]. Additionally, refinements in technology and operative techniques have improved esthetic outcomes while minimizing complications. These advancements include more conservative mastectomies with thicker mastectomy flaps, acellular dermal matrices, and fat grafting [7]. Despite such improvements, the overall mechanisms behind tissue expanders remain similar to the Radovan’s technique. The traditional saline expanders require periodic bolus percutaneous needlesticks injections during the postoperative period, associated with discomfort, multiple outpatients visits, days out from work and potentially the risk of microbial contamination during repeated injections [8–14].

Recently, other filling options such as air or carbon dioxide (CO2) have been reported as alternative fill media for tissue expanders [10, 11]. Various methods have been described for breast reconstruction, encompassing the AeroForm tissue expander system (AirXpanders, San Francisco, CA), which expands using CO2 from an internal container, tissue expanders initially filled with air and later with saline, and tissue expanders filled with saline only [12, 13]. These strategies aim to achieve the desired volume minimizing patients’ discomfort by reducing the need for ambulatory visits, accelerating the expansion period, and potentially reducing complications. Indeed, it has been hypothesized that because of gravitational pull, saline accumulates at the bottom of the tissue expander, creating more tension at the lower pole of the mastectomy skin flaps, skin necrosis, and expander extrusion compared to air-filled expanders [14]. However, the safety and the potential benefit of filling breast tissue expanders with air rather than saline has not been fully analyzed yet. The aim of this systematic review was to qualitatively and quantitatively synthetize the available evidence on the topic.

## Materials and Methods

A systematic review and meta-analysis were performed and reported according to the Preferred Reporting Items for Systematic Reviews and Meta-Analysis (PRISMA) guidelines [15]. Institutional review board approval and informed consents were not required for this study, since all the reported data were obtained from the available published literature.

### Inclusion and Exclusion Criteria

The PICOS framework [16] was used in developing the literature search strategy:

- *Population* (P): adult patients undergoing two-stage implant-based breast reconstruction;
- *Intervention* (I): saline expander placement and filling;
- *Comparator *(C): air expander placement and filling;
- *Outcome *(O): rate of complications and time to final expansion;
- *Study type *(S): Randomized controlled trials, prospective and retrospective cohort studies with a control group.

Studies were excluded if: (a) they were not in English, (b) they were not available in full-text form, (c) data on complications or time to final expansion were not extractable, (d) the article type was a conference abstract, review, case report, book chapter or letter to the editor, (e) data presented were not specific to breast reconstruction, (f) they did not include a control group. All articles had to be published in a peer-reviewed journal, but no restriction on publication date was applied.

### Outcome Measures

The primary outcomes were the risk ratio of overall complications and the mean difference in time to expansion between saline and air-filled breast expanders. Secondary outcomes were rates of mastectomy flap necrosis, hematoma, seroma, infection, expander exposure or extrusion, and readmission. Mastectomy flap necrosis was defined as any epidermolysis or partial or full thickness necrosis of the nipple-areolar complex or mastectomy skin flaps requiring non-operative or operative management. Hematoma was defined as any postoperative collection of blood recognized during physical examination or ultrasound. Seroma was defined as any serous fluid collection identified during physical examination or ultrasound after drain removal regardless of whether it required aspiration or drainage. Infection was defined as any infection recognized clinically by the surgeon, regardless of positive cultures or infection requiring intravenous or oral antibiotics with or without surgical intervention. Expander exposure or extrusion was defined as the dehiscence of the skin with exposure of the tissue expander requiring explantation. Readmission was defined as 30 days of unplanned readmission after tissue expander placement.

### Data Source and Study Search

An electronic search was performed on PubMed, Embase, and Cochrane Library using relevant keywords, phrases, and medical subject headings (MeSH) terms. The search strategy applied for PubMed was: ("air"[MeSH Terms] OR"air"[All Fields] OR ("saline solution"[MeSH Terms] OR ("saline"[All Fields] AND"solution"[All Fields]) OR"saline solution"[All Fields] OR"saline"[All Fields] OR"salines"[All Fields]) OR ("liquid"[All Fields] OR"liquid s"[All Fields] OR"liquids"[All Fields]) OR ("fluid"[All Fields] OR"fluid s"[All Fields] OR"fluids"[All Fields])) AND ("expander"[All Fields] OR"expanders"[All Fields])

The reference list of each selected article was checked to screen for potentially relevant studies (*i.e.*, snowballing method). The last search was carried out on November 26, 2023.

### Selection of Studies and Data Extraction

Two reviewers independently conducted the electronic literature search (E.B. and G.G.). The reference lists from 3 databases (*i.e.*, PubMed, Embase, and Cochrane Library) were merged and the duplicates removed using the reference management software EndNote^®^ X9 (version X9.3.3). Titles and abstracts of unresolved papers were screened. Whenever appropriate, full texts of relevant articles underwent subsequent evaluation for eligibility. Discrepancies were resolved by a third author (M.A.B.). Data extracted from selected articles were archived in a customized Excel® (Microsoft Corp, Seattle, Washington, USA) spreadsheet.

### Data Synthesis and Statistical Analysis

The analysis was performed using R software for statistical computing (R 4.0.1; “meta” package). Data were pooled using a fixed or a random effects model according to the identified level of heterogeneity, following the recommendation of the Cochrane Handbook for Systematic Reviews of Interventions [17]. The mean difference (MD) was calculated as a measure of effect size to compare continuous variables, while risk ratio (RR) was calculated for dichotomous variables. All results were expressed with a 95% confidence interval (CI). A forest plot graph was created for each outcome. Statistical significance was defined as *p* < 0.005.

To assess heterogeneity among studies, the forest plots of study outcomes were examined to analyze the level of consistency considering the size and the direction of effects [18]. In addition, we calculated the I^2^ statistic to quantify heterogeneity, assuming values >50% as indicative of substantial heterogeneity [18]. Cochrane Q test was also analyzed as the I^2^ statistics were underpowered in the presence of a low number of included studies [19]. Specifically, p < 0.05 was considered to indicate the statistical significance of the Q test. The maximum-likelihood estimator was used to estimate the between-study variance (τ^2^) [20]. Analysis of publication bias was performed by inspection of the funnel plot, and calculating Peters’ linear regression test, which statistically examines the asymmetry of the funnel plot [20]. If I^2^ statistics was > 50% or Q statistics resulted in *p* < 0.05, a more conservative random effect model was used. If not, a fixed effects model was used.

### Risk of Bias and Study Quality Assessment

The methodological quality of included studies was assessed independently by two separate authors (E.B. and E.D.). Since no RCTs were included, the Methodological Index for Non-randomized Studies (MINORS) criteria, a validated instrument designed to assess the methodological quality of non-randomized studies, was used to measure bias.^21^ The maximum MINORS score for comparative studies is 24 [21].

## Results

### Electronic Database Search and Key Characteristics of Included Studies

**Fig. 1:**
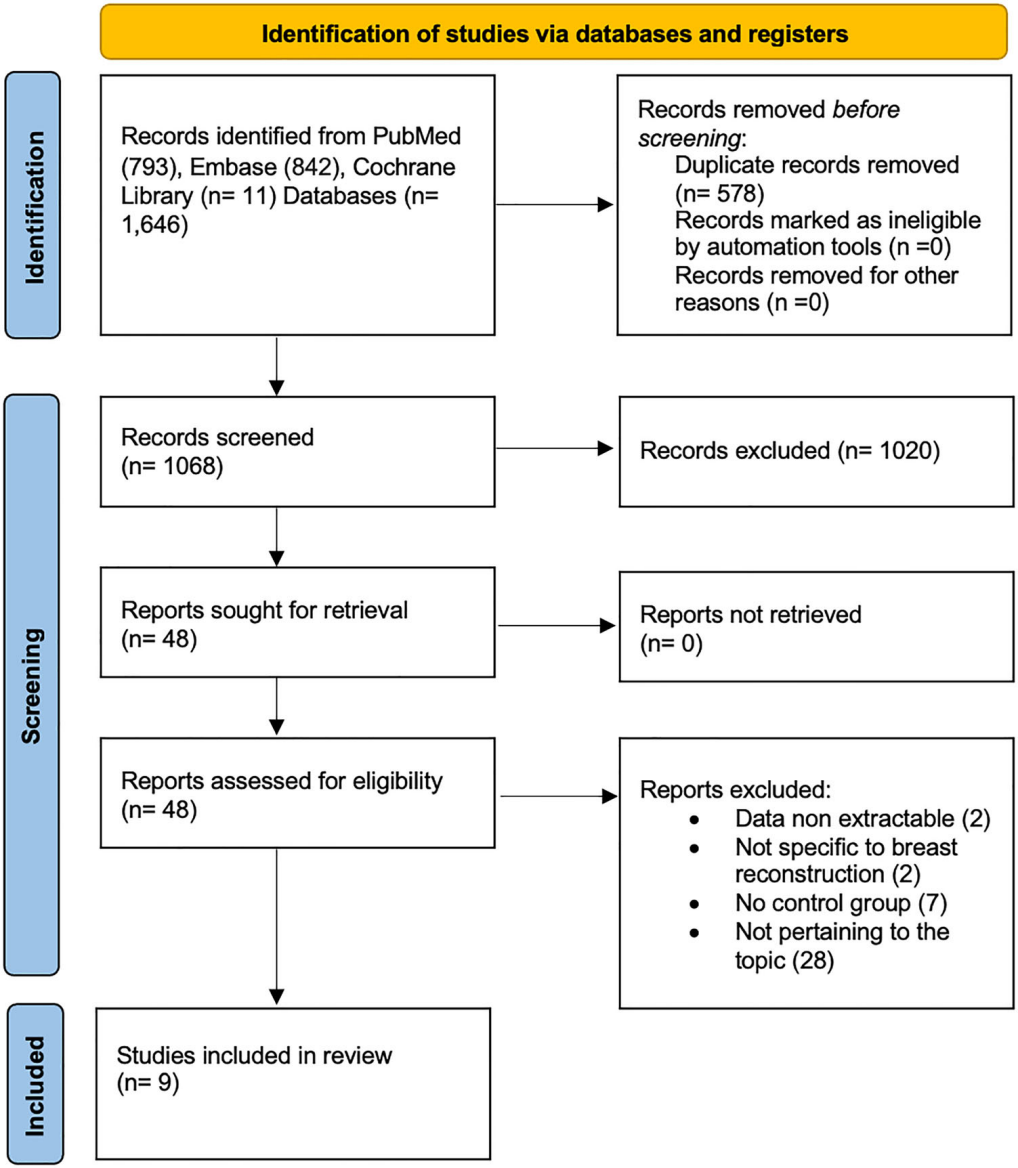
PRISMA flow diagram

From the initial search, a total of 1,646 eligible papers were identified. After eliminating duplicate entries and screening titles and abstracts, 48 full-text manuscripts were evaluated for eligibility. Upon applying inclusion and exclusion criteria, nine articles were included in both qualitative and quantitative synthesis [14, 22–29]. The PRISMA flow diagram is shown in Fig. 1. Characteristics of the articles included in this study are shown in Table 1.

**Table 1:** Studies’ general characteristics

Author	Year	Study design	Type of expander	Saline-filled expanders								Air-filled expanders							
				N of patients	N of breasts	Unilateral	Bilateral	Age (SD)	BMI (SD)	N of active smokers	N of diabetic patients	N of patients 2	N of breasts 2	Unilateral 2	Bilateral 2	Age (SD) 2	BMI (SD) 2	N of active smokers 2	N of diabetic patients 2
Ascherman et al.	2016	RCT	Aeroform	52	88	16	36	51,1 (9,9)	24,8 (4,25)			98	168	28	70	48,9 (9,83)	25,2 (4,08)		
Bae et al.	2022	RC	Air	257	282	232	25	44,7 (7,1)	22,2	2	4	143	161	125	18	45,4 (7,6)	22,7	1	2
Bamba et al.	2022	RC	Air	25	44			46,6 (10,3)	28,5 (6,8)	0	3	28	44	44		48,6 (10,8)	29,2 (5,8)	1	5
Chopra et al.	2018	RC	Aeroform	68	111			50 (10,5)	27 (6)	3	10	47	74			49,1 (12,6)	27 (6)	3	1
Plotsker et al.	2022	RC	Air	188	305	71	117			3	9	372	623	121	251			9	16
Porter et al.	2019	RC	Air		282			51,2 (11,5)	27,22 (6,07)	9			21			51 (9,3)	23 (2,87)	0	
Sergesketter et al.	2023	RC	Air	377	588	132	228	52,9 (11,2)	26,4 (5,1)	59	12	130	172	44	64	52,4 (11,9)	27,1 (5,1)	9	2
Yesantharao et al.	2021	RC	Air	29	47	11	18	48 (13,4)	24 (5)	1	1	58	97	19	39	48,6 (9,9)	23,8 (9,8)	1	4
Zeidler et al.	2014	RCT	Aeroform	24	39	9	15					58	97	19	39				

The included studies encompassed a total of 1954 patients and 3243 breasts. Specifically, 1020 patients accounting for 1786 breasts were reconstructed using saline expanders, while 934 patients and 1457 breasts reconstructed using expanders filled with air or AeroForm tissue expanders were included.

In the saline group, the mean age was 48.9 years [95% CI: 45.6; 52.3], and the mean BMI was 26.0 [95% CI: 23.9; 28.0]. In the air group, the mean age was 48.7 years [95% CI: 46.2; 51.2], and the mean BMI was 26.4 [95% CI: 24.1; 28.8]. No differences between the saline group and the air group were found in terms of mean age (*p*=0.91) and mean BMI (p=0.19). No difference between the two groups was observed in the number of unilateral (*p*=0.27) and bilateral (*p*=0.60) breast reconstructions. Additionally, the two groups did not differ in terms of mastectomy types: simple mastectomy (*p*= 0.73), skin-sparing mastectomy (*p*=0.24), nipple-sparing mastectomy (*p*= 0.44), lumpectomy (*p*= 0.07), other mastectomy types (*p*= 0.31). Notably, no between-group difference was found in pre- and subpectoral placement (*p*=0.39), use of acellular dermal matrices (*p*=0.14), and mastectomy weight (*p*=0.98). Tissue expander size was greater in the air group with a MD of 19.55 cc [95% CI: 7.39; 31.71, *p*=0.001]; however, there was no difference in the initial filled volume (*p*=0.79).

The groups were homogenous in terms of oncological treatments: postoperative radiation therapy (*p*=0.72), neoadjuvant (*p*=0.14), and adjuvant chemotherapy (*p*=0.50).

There was no difference between the two groups in the number of active smokers (RR=0.66 [95% CI: 0.39; 1.09], *p*=0.10) and patients with diabetes (RR=0.74 [95% CI: 0.44; 1.24], *p*=0.25). Other comorbidities were seldom reported.

### Risk of Bias Assessment

In the nine included studies, MINORS scores ranged from 13 to 21, with a mean of 17.3. The major deficiency was lack of prospective collection of data. All studies showed a clearly stated aim, appropriate endpoints, scarce loss to follow-up and appropriate baseline equivalence of groups as well as statistical analysis, as seen in Supplement 1.

### Total Complications

Complications are summarized in Table 2.

**Table 2:** Complications reported in each of the included studies

Author	Saline-filled expanders										Air-filled expanders									
	N of breasts	N of complications	N of reoperations	N of flap necrosis	Hematoma	Seroma	Infection	Expander rupture	Tissue expander exposure/extrusion	Readmission	N of breasts	N of complications	N of reoperations	N of flap necrosis	Hematoma	Seroma	Infection	Expander rupture	Tissue expander exposure	Readmission
Ascherman et al.	88	33	NS	NS	3	5	6	NS	1	NS	168	63	NS	NS	2	15	9	NS	2	NS
Bae et al.	282	74	NS	64	7	NS	7	NS	3	11	161	33	NS	24	6	NS	6	NS	1	6
Bamba et al.	44	7	NS	0	0	NS	NS	NS	NS	NS	44	NS	NS	2	0	NS		NS	NS	NS
Chopra et al.	111	51	NS	15	4	10	6	1	3	1	74	24	NS	6	0	4	0	0	1	0
Plotsker et al.	305	54	NS	4	6	18	29	NS	4	NS	623	140	NS	15	15	61	45	NS	17	NS
Porter et al.	282	291	NS	12	6	43	63	6	62	75	21	15	NS	0	1	5	3	0	3	3
Sergesketter et al.	588	233	32	63	7	38	70	NS	3	20	172	79	13	21	3	8	20	NS	5	9
Yesantharao et al.	47	17	9	7	0	0	8	NS	2	NS	97	19	7	10	1	2	9	NS	7	NS
Zeidler et al.	39	NS	NS	NS	NS	NS	NS		NS	NS	97	2	NS	NS	NS	NS	NS	NS	NS	NS

**Fig. 2:**
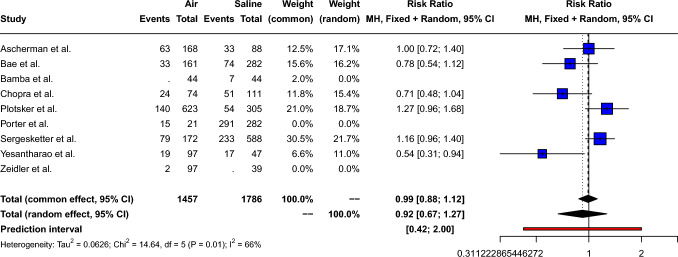
Forest plot showing the risk ratio of total complications

The pooled risk ratio of total complications for air-filled compared to saline-filled breast expanders was 0.92 [95% CI: 0.67; 1.27, *p*=0.53], as shown in Fig. 2. High between-study heterogeneity (Q=14.6, *p*=0.01) was measured: *I*^2^= 65.8% [95% CI: 18.2%; 85.7%] τ^2^= 0.06. Therefore, a random effect model was used. Peters’ linear regression test showed possible publication bias (t= − 3.33, *p*=0.03); however, visual inspection of the funnel plot shows a symmetric distribution of the points, as shown in Supplement 2.

### Mastectomy Flap Necrosis

**Fig. 3:**
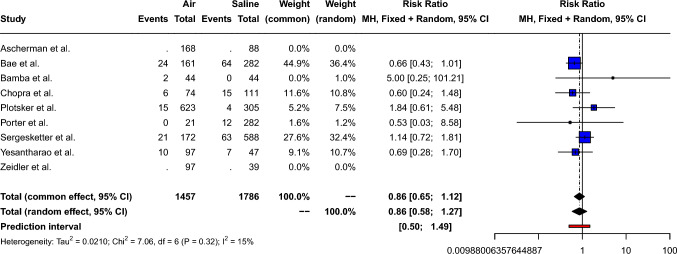
Forest plot showing the risk ratio of mastectomy flap necrosis

The pooled risk ratio of the skin flap necrosis after mastectomy for air-filled compared to saline-filled breast expanders was 0.86 [95% CI: 0.65; 1.12, *p*=0.26], as shown in Fig. 3. Small between-study heterogeneity (Q=7.1, *p*=0.32) was measured: *I*^2^= 15.0% [95% CI: 0.0%; 58.7%] τ^2^= 0.02. Therefore, a fixed effect model was used. Peters’ linear regression test showed no obvious publication bias (t=−0.71, *p*=0.51) and visual inspection of the funnel plot shows a symmetric distribution of the points, as shown in Supplement 3.

### Hematoma

**Fig. 4:**
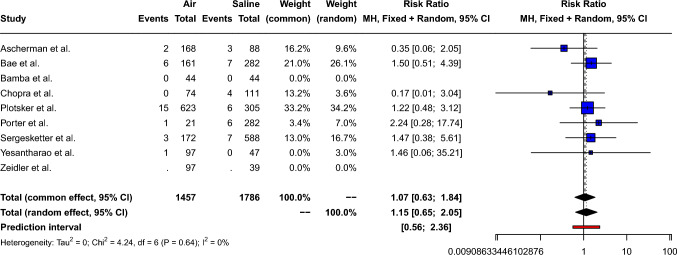
Forest plot showing the risk ratio of hematoma

The pooled risk ratio of postoperative hematoma for air-filled compared to saline-filled breast expanders was 1.07 [95% CI: 0.63; 1.84, *p*=0.80], as shown in Fig. 4. Small between-study heterogeneity (Q=4.24, *p*=0.64) was measured: *I*^2^= 0.0% [95% CI: 0.0%; 70.8%] τ^2^= 0. Therefore, a fixed effect model was used. Peters’ linear regression test showed no obvious publication bias (t=−1.52, *p*=0.46) and visual inspection of the funnel plot shows a symmetric distribution of the points, as shown in Supplement 4.

### Seroma

**Fig. 5:**
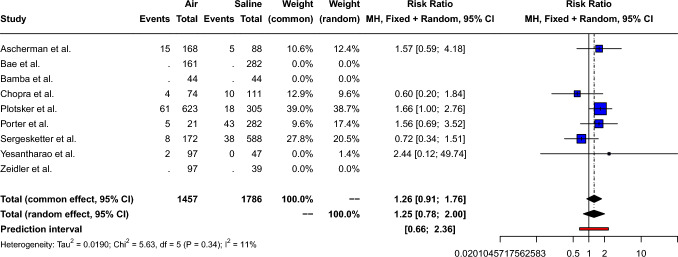
Forest plot showing the risk ratio of seroma

The pooled risk ratio of postoperative seroma for air-filled compared to saline-filled breast expanders was 1.26 [95% CI: 0.91; 1.76, *p*=0.16], as shown in Fig. 5. Small between-study heterogeneity (Q=5.63, *p*=0.34) was measured: *I*^2^= 11.2% [95% CI: 0.0%; 77.5%] τ^2^= 0.02. Therefore, a fixed effect model was used. Peters’ linear regression test showed no obvious publication bias (*t*= − 0.17, *p*=0.88) and visual inspection of the funnel plot shows a symmetric distribution of the points, as shown in Supplement 5.

### Infection

**Fig. 6:**
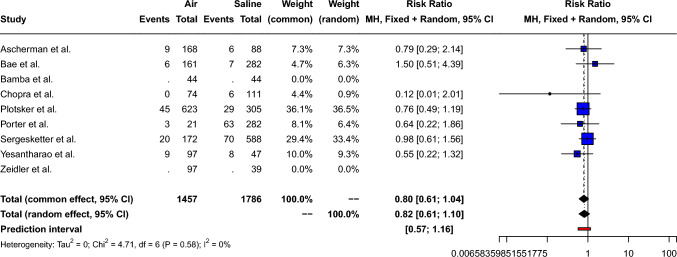
Forest plot showing the risk ratio of infection

The pooled risk ratio of postoperative infection for air-filled compared to saline-filled breast expanders was 0.80 [95% CI: 0.61; 1.04, *p*=0.09], as shown in Fig. 6. Small between-study heterogeneity (Q=4.71, *p*=0.58) was measured: *I*^2^= 0.0% [95% CI: 0.0%; 70.8%] τ^2^= 0.02. Therefore, a fixed effect model was used. Peters’ linear regression test showed no obvious publication bias (*t*= − 1.55, *p*=0.18) and visual inspection of the funnel plot shows a symmetric distribution of the points, as shown in Supplement 6.

### Tissue Expander Extrusion or Exposure

**Fig. 7:**
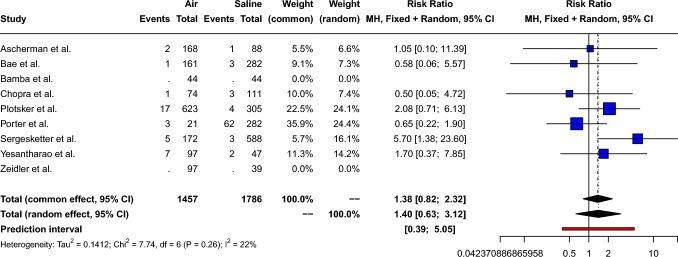
Forest plot showing the risk ratio of expander extrusion/exposure

The pooled risk ratio of postoperative extrusion or exposure for air-filled compared to saline-filled breast expanders was 1.38 [95% CI: 0.82; 2.32, *p*=0.23], as shown in Fig. 7. Small between-study heterogeneity (Q=7.74, *p*=0.26) was measured: *I*^2^= 22.5% [95% CI: 0.0%; 65.4%] τ^2^= 0.14. Therefore, a fixed effect model was used. Peters’ linear regression test showed no obvious publication bias (*t*= − 1.0, *p*=0.36) and visual inspection of the funnel plot shows a symmetric distribution of the points, as shown in Supplement 7.

### Need for Readmission

**Fig. 8:**
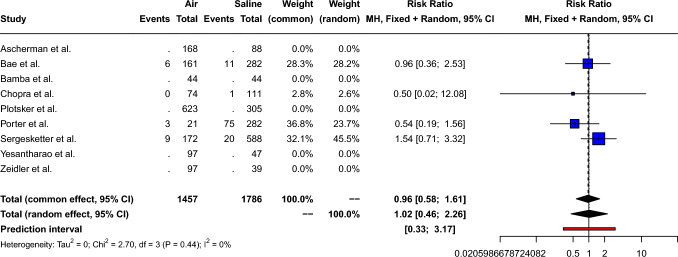
Forest plot showing the risk ratio of unplanned readmission

The pooled risk ratio of readmission for air-filled compared to saline-filled breast expanders was 0.96 [95% CI: 0.58; 1.60, *p*=0.88], as shown in Fig. 8. Small between-study heterogeneity (Q=2.70, *p*=0.44) was measured: *I*^2^= 0.0% [95% CI: 0.0%; 84.7%] τ^2^= 0. Therefore, a fixed effect model was used. Peters’ linear regression test showed possible publication bias (*t*= − 6.52, *p*=0.02); however, visual inspection of the funnel plot shows a symmetric distribution of the points, as shown in Supplement 8.

#### Time to Expansion

**Fig. 9:**
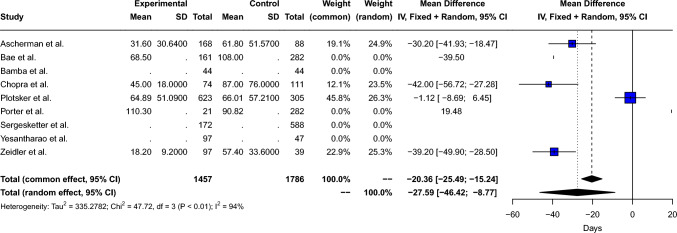
Forest plot showing the mean difference of time to expansion

The mean difference in days needed to achieve final expansion between air-filled and saline-filled breast expanders was − 27.59 [95% CI: − 46.42; − 8.78, *p*=0.004], as shown in Fig. 9. High between-study heterogeneity (Q=47.72, *p*<0.001) was measured: *I*^2^= 93.7% [87.1%; 96.9%], τ^2^= 335. Therefore, a random effect model was used. Peters’ linear regression test showed possible publication bias (*t*= − 6.52, *p*=0.02); however visual inspection of the funnel plot shows a symmetric distribution of the points, as shown in Supplement 9.

## Discussion

**Fig. 10:**
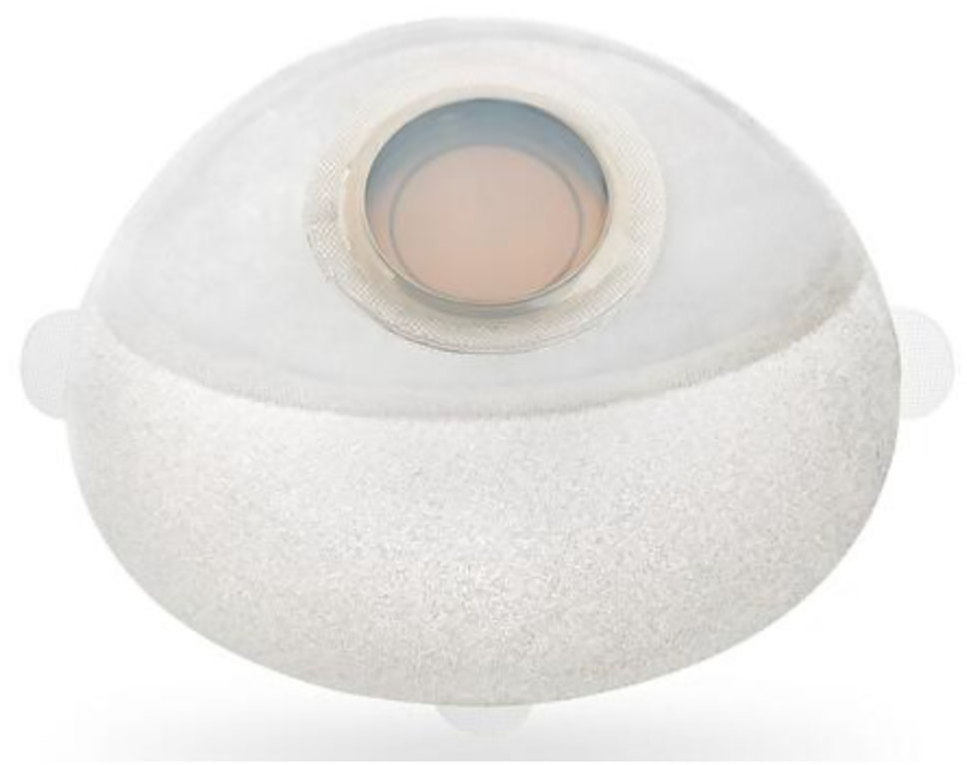
A Mentor (Mentor Worldwide LLC, Irvine, CA) tissue expander used in two-stage breast reconstruction. The device consists of a silicone shell with an integrated injection port, allowing gradual saline inflation over time to create space for a permanent implant. Optional suture tabs at 3, 6 and 9 o’clock positions can help stabilize the expander location

**Fig. 11:**
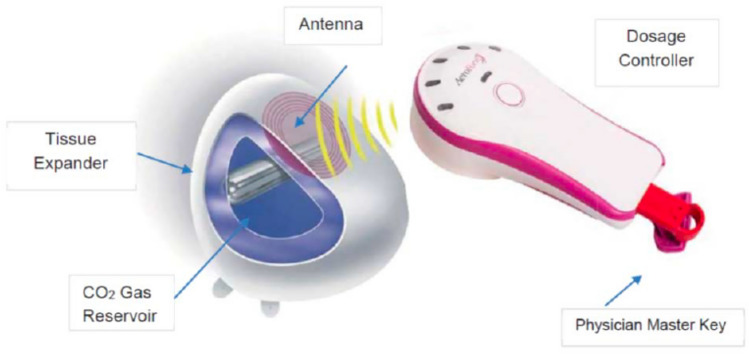
Aereoform (AirXpanders, San Francisco, CA) is a carbon dioxide gas controlled tissue expander with an outer textured silicone shell and a non-distensible inner bag which are anatomically shaped to allow for directional expansion in the lower, anterior pole. The AeroForm Tissue Expander contains a reservoir of compressed carbon dioxide, which is released within the AeroForm Tissue Expander by using the Dosage Controller. A receiving antenna and electronics within the AeroForm Tissue Expander enable communication with the Dosage Controller. The AeroForm Tissue Expander has no intrinsic electrical power, batteries, or software, and can only be activated by the Dosage Controller (https://www.accessdata.fda.gov/cdrh_docs/reviews/den150055.pdf)

Two-stage tissue expander-to-implant procedure is a common strategy in breast reconstruction [30–32]. In 2022, according to the American Society of Plastic Surgeons, two-stage alloplastic breast reconstruction with tissue expander was performed in 54,5% of total breast reconstruction procedures [33]. The use of a tissue expander (Fig. 10) is generally preferred in patients with insufficient skin for direct-to-implant reconstruction or in cases deemed at high risk of mastectomy skin flap necrosis [7]. The main complications associated with a two-stage breast reconstruction include mastectomy skin flap necrosis, infection, tissue expander extrusion, hematoma, and seroma [22, 34, 35]. However, the two-stage breast reconstruction process also poses challenges in terms of both logistical organization and patient care management, such as the requirement for a subsequent surgical procedure, an extended follow-up period, frequent outpatient visits, delayed achievement of the final result and patient’s satisfaction [36]. Moreover, patient’s discomfort and anxiety are often exacerbated by the need for periodic needle injections to fill the expander which are also associated with potential risks of infection and expander puncture. This lengthy process can hinder women’s daily activities and discourage patients from choosing to undergo breast reconstruction [27, 28]. Therefore, it is important to identify new strategies to improve outcomes and reduce the burden for women receiving a two-stage breast reconstruction. Recently, shifting the expansion medium to air rather than saline has been reported as a possible option to accelerate the reconstructive journey, reduce the burden associated with a two-stage reconstruction and ideally minimize complications [22, 27]. Indeed, the hypothesized rationale behind air expansion in breast reconstruction is to improve outcomes by exerting less gravitational pull on healing tissues, reducing mechanical stress and postoperative discomfort. Its physical properties as well as device’s features allow for faster expansion with fewer outpatient visits [11, 14]. Six studies compared saline with air expansion while three compared saline with carbon dioxide (CO2)-based tissue expansion. The CO2-filled breast expander is named Aeroform (AirXpanders, Inc., San Jose, Calif.) (Fig. 11). It is a device that allows for gradual and needle-free expansion at home with a handheld remote patient controller and contains a reservoir of compressed carbon dioxide. The controller communicates wirelessly to release an amount of 10 ml of CO2 per dose with a maximum of three patient-initiated expansions per day [28]. Some authors advocate that filling with CO2 has several clinical benefits such as the ability to expand gradually in less time, at home (depending on the patient’s level of comfort) and it minimize the risk of iatrogenic introduction of bacteria into the implant pocket or expander puncture [27]. However, the Aeroform expander has potential adverse effects related to device (i.e., device communication failure), and deflation after the expansion is not allowed. Additionally, there is a paucity of data documenting the safety and efficacy of air-filled expander reconstructions in patients undergoing radiation therapy. Indeed, air has different physical properties compared to saline, which closely resembles tissue in terms of radiation interactions. Therefore, air-filled expanders may impact radiation therapy planning and delivery [37]. However, according to available literature, filling with air has demonstrated both safety and efficacy.

Chopra et al. [27]. found that Aeroform positioned in the prepectoral space had a statistically significant reduction in infections and readmission for IV antibiotics compared to saline-filled expanders. They also demonstrated a significant decrease in days to complete expansion and consequently days to complete reconstruction [27]. J. Bae et al. [23]. included patients who underwent immediate subpectoral tissue expander reconstruction. The air-filled group showed a significantly lower rate of mastectomy flap necrosis, a shorter period to complete expansion, and a reduction in the number of visits for expansion. They hypothesized that the air expanders allow a more effective expansion of the mastectomy skin flap during the immediate postoperative period, resulting in faster time to complete expansion [23]. However, four of the nine included articles demonstrated no statistical evidence of complications reduction in air-filled expanders [24–26, 28]. E. Plotsker et al. [24]. demonstrated, after a propensity matching, no significant advantage of air-filled expanders in preserving mastectomy skin flap viability and in patient-reported outcomes.

The results of our meta-analysis corroborate the lack of significant difference between the two filling media on the rate of complications in two-stage expander reconstruction. According to the available literature, air-filled expanders were not able to reduce the rate of mastectomy flap necrosis, infection, expander exposure, hematoma, seroma, and readmission compared to saline expanders. However, air-filled breast expanders we found to reduce the time to expansion compared to saline-filled breast expanders with a mean reduction of 27.59 [CI: − 46.42; − 8.77] days in air versus saline expanders. However, further evidence is necessary to confirm the findings obtained in this study.

The main strength of this study is represented by the similar characteristics of the populations under investigation in terms of demographics, comorbidities, oncological therapies, and surgical details. However, heterogeneity in the types of expanders, time of filling and follow-up may have introduced bias in the analysis. Additionally, heterogeneity in prepectoral and submuscular placement of the tissue expander was observed. Sub-analysis on potential predictive factors of complications could not be performed due to unreported data and further research is needed to identify which category of patients could benefit more from air-filled expanders, cost-effectiveness, and patient-reported outcomes.

## Conclusions

Air-filled expanders represent an alternative reconstructive option in the field of two-stage breast reconstruction. Despite limited by the only initial available evidence, they appeared to be safe and associated with a similar risk of complications compared to saline-filled expanders. However, they may enable faster postoperative expansion and fewer outpatient expansion visits compared to saline-filled expanders. However, additional prospective controlled studies with larger samples are necessary to verify the safety of the device and its interaction with oncological therapies.

## Supplementary Information

Below is the link to the electronic supplementary material.

Supplementary file1 (DOCX 18 KB)

Supplementary file2 (DOCX 129 KB)

## Declarations

### Human or Animals Participants

This article does not contain any studies with human participants or animals performed by any of the authors.
