# Supplementary material for: Outcomes of Air Versus Saline-filled Breast Expanders: A Systematic Review and Meta-analysis
Source: Aesthetic Plast Surg. 2025 May 23;49(21):6025–37. doi: 10.1007/s00266-025-04918-5 (PMC12705835; doi:10.1007/s00266-025-04918-5)
Supplement: Supplementary file 1 — Supplementary file1 (DOCX 18 KB) [file 266_2025_4918_MOESM1_ESM.docx]

| **Authors** | ***Clearly stated aim*** | ***Inclusion of consecutive patients*** | ***Prospective collection of data*** | ***End points appropriate to aim of study*** | ***Unbiased assessment of the study  end point*** | ***Follow up period appropriate to aim of study*** | ***Loss to follow up <5%*** | ***Prospective calculation of study size*** | **An adequate control group**: having a gold standard diagnostic test or therapeutic intervention recognized as the optimal  intervention according to the available published data | **Contemporary groups**: control and studied group should be managed during the same time period (no historical comparison) | **Baseline equivalence of groups**: the groups should be similar regarding the criteria other than the studied endpoints. Absence  of confounding factors that could bias the interpretation of the results | **Adequate statistical analyses**: whether the statistics were in accordance with the type of study with calculation of confidence  intervals or relative risk | ***MINORS score*** |
| --- | --- | --- | --- | --- | --- | --- | --- | --- | --- | --- | --- | --- | --- |
| Ascherman et al. | 2 | 0 | 2 | 1 | 1 | 1 | 2 | 2 | 2 | 2 | 2 | 2 | 19 |
| Bae et al. | 2 | 2 | 0 | 2 | 1 | 1 | 2 | 0 | 2 | 1 | 2 | 2 | 17 |
| Bamba et al. | 2 | 0 | 0 | 2 | 1 | 1 | 2 | 0 | 2 | 2 | 2 | 2 | 16 |
| Chopra et al. | 2 | 2 | 0 | 2 | 1 | 2 | 2 | 0 | 2 | 2 | 2 | 2 | 19 |
| Plotsker et al. | 2 | 0 | 0 | 2 | 2 | 2 | 2 | 0 | 2 | 1 | 2 | 2 | 17 |
| Porter et al. | 2 | 0 | 0 | 2 | 1 | 0 | 2 | 0 | 1 | 1 | 2 | 2 | 13 |
| Sergesketter et al. | 2 | 1 | 0 | 2 | 2 | 1 | 2 | 0 | 2 | 1 | 2 | 2 | 17 |
| Yesantharao et al. | 2 | 0 | 0 | 2 | 1 | 2 | 2 | 0 | 2 | 2 | 2 | 2 | 17 |
| Zeidler et al. | 2 | 0 | 2 | 2 | 2 | 1 | 2 | 2 | 2 | 2 | 2 | 2 | 21 |

**Supplement 1.** MINORS scores of included studies.
