# Supplementary material for: Outcomes of Air Versus Saline-filled Breast Expanders: A Systematic Review and Meta-analysis
Source: Aesthetic Plast Surg. 2025 May 23;49(21):6025–37. doi: 10.1007/s00266-025-04918-5 (PMC12705835; doi:10.1007/s00266-025-04918-5)
Supplement: Supplementary file 2 — Supplementary file2 (DOCX 129 KB) [file 266_2025_4918_MOESM2_ESM.docx]

Supplements

Supplement 2: Funnel plot for total complications

Supplement 3: Funnel plot for mastectomy flap necrosis


Supplement 4: Funnel plot for hematoma

Supplement 5: Funnel plot for seroma

Supplement 6: Funnel plot for infection

Supplement 7: Funnel plot for extrusion

Supplement 8: Funnel plot for re-admission

Supplement 9: Funnel plot for time-to-expansion
